# The relationship of internalizing problems with emotional intelligence and social skills in secondary education students: gender differences

**DOI:** 10.1186/s41155-018-0115-y

**Published:** 2019-01-25

**Authors:** Carlos Salavera, Pablo Usán, Pilar Teruel

**Affiliations:** 1 Research Group OPIICS, University of Zaragoza, Zaragoza, Spain; 2 Faculty of Education, University of Zaragoza, c/ Pedro Cerbuna, 12, 50009 Zaragoza, Spain

**Keywords:** Internalizing problems, Social skills, Emotional intelligence, Secondary education students

## Abstract

Internalizing problems (depression, anxiety, social anxiety, somatic complaints, post-traumatic symptoms, and obsession-compulsion) are very important in adolescents’ development. These problems can be related with people who lack social skills and poorly handle their emotions. This study assessed 1358 secondary education students (12–17 years) to analyze the relationship linking internalizing problems, emotional intelligence, and social skills. The results showed not only how these constructs were related, but how students’ internalizing problems varied according to their emotional intelligence and social skills. They also indicated that two in every three males, and just over one in every two females, obtained high scores for internalizing problems. The model showed a good fit: *χ*^2^(85) = 201.161 *p* < 0.001; *χ*^2^/*gl* = 2.367; CFI = 0.919; NFI = 0.869; TLI = 0.900; RMSEA = 0.075, IC 95% (0.062–0.089). Finally, gender influenced the way that internalizing problems, emotional intelligence, and social skills were related, and an inverse relation appeared to link internalizing problems, emotional intelligence (*r* = − .77), and social skills (*r* = − .52) for females, while this relationship was poorer for males. By way of conclusion, we state that internalizing problems are related with emotional intelligence and social skills in secondary education students, but this relationship differs according to gender.

## Background

Adolescence is a transition period, a growth cycle which announces that infancy is ending. In this stage, adolescents search for their real selves and their identity, shift from individual trends to group trends, seek uniformity in their conduct, and wish to control these many changes. In parallel, they have to adapt to many major physical, intellectual, social, and emotional changes and start to experience the need for social acceptance, identification, and affect from peers. Adolescence is perhaps in emotional and development terms the most complicated stage in the whole life cycle (Livesey & Ronstain, 2017).

The adolescent begins two important processes: searching for self-knowledge and achieving autonomy. A good realization of these processes helps people better cope with stress in this stage, which leads to a greater perception of happiness (Salavera, Usán, Pérez, Chato, & Vera, 2017). Accordingly, self-perception, social skills, and emotional functioning are fundamental. Adolescence is characterized by the development of emotional intelligence (ability to manage or self-regulate emotions) and social competence (ability to interact effectively with others). The adolescent moves from concrete thinking to abstract thinking and begins to develop more advanced reasoning skills. This development facilitates progressive autonomy, contributes to young people’s well-being and psychosocial development, and protects them from developing psychological problems in the face of stressful life experiences, where personal development, adequate social skills, and good emotional functioning are fundamental for adolescents (Gaete, 2015; Salavera, Usán, & Jarie, 2017).

## Problems in adolescence

Adolescents’ problems are divided into the following two groups: (1) internalizing problems, which basically include the typical emotional alterations of anxiety disorders and depression (anxiety, depression, somatic complaints, post-traumatic disorders, obsession-compulsion, etc.) and (2) externalizing problems, related with disruptive behavior that causes conflicts in relationships with others (conduct disorders, defiant conduct, aggression, controlling rage, etc.).

The literature outlines differences between males and females in the prevalence of different psychological disorders in infancy and adolescence (Crick & Grotpeker, 1995; Ortuño-Sierra, Fonseca-Pedrero, Sastre, & Muñiz, 2017). For instance, males are two to four times more likely to present conduct problems (Campos, Besser, Morgado, & Blatt, 2014; Navarro-Pardo, Meléndez, Sales, & Sancerni, 2012), while females present more emotional problems (Chaplin & Aldao, 2013; Schäfer, Naumann, Holmes, Tuschen-Caffier & Samson, 2017).

Both externalizing and internalizing problems are related with children’s adaptation to their main socialization contexts (family, school, and classmates), and they strongly impact adolescents’ lives.

One of the highest prevalence rates of internalizing problems is found in adolescents and affect 10% and 20% of the general population. Youths soon grasp the reality around them by occasionally configuring those who present internalizing problems, a threatening dimension that deals with personal, family, school, and social relationships. When faced with such situations, adolescents display fearful and anxious reactions that are merely physiological defense reactions to potential dangers, and which place them in a state of alert to possible threats.

These problems are directly related with adolescents’ habitual setting (family, school, and classmates), and they affect adolescents as they see that their relationships with family or peers are altered (Jaccar, 2016). Some consequences are social isolation, worse academic performance, or obtaining academic marks below those expected for what they are capable of. When these problems continue over time, the result may be academic failure, which can have implications for adolescents and their surroundings. In Spain, almost 40% of students reach the last compulsory secondary education course having accumulated some degree of delay (Fernández, Mena, & Riviere, 2010).

## Internalizing problems and social skills

Other variables may somehow come into play in these internalizing problems as protectors or risk factors. This may occur with social skills and emotional intelligence (EI), where the absence of the former or the poor handling of the latter can alter someone’s reality. Social skills are a series of conducts required to effectively and satisfactorily interact and relate with others that can show the capacity to behave in a way that is rewarded and to avoid behaving in a way that is punished or ignored by others (Kinnaman & Bellack, 2012). In other words, they are a series of conducts displayed by individuals in an interpersonal context to express feelings, attitudes, desires, opinions, or rights adequately for a given situation by respecting these conducts in others, which generally solve the immediate problems in a given situation, and reduce the likelihood of future problems (Caballo, 2002; Was & Warneken, 2017). These skills are acquired mainly by learning through observation, imitation, testing, and information and are learned conducts as someone is not born with a given repertoire of social skills, but people include them while they develop, learn, and grow. There are two types of social skills: basic and complex. The former has to be learned to develop the latter. This process begins in infancy and develops basically in adolescence to confer someone communicative and relational tools in adulthood, and they cover the need to establish positive social relationships as a source of satisfaction and personal well-being (Lent et al., 2017).

Internalizing problems include controlled behavior directed mainly toward oneself and creating difficulties for the person presenting them, hence the importance in their relationship with social skills (Tandon, Cardeli, & Luby, 2009). Internalizing problems present three interrelated aspects: at the cognitive level (irrational negative thoughts), at the physical level with the presence of tension symptoms (fears), and at the behavioral level (deficit of social skills). People with internalizing problems show considerable inhibition, difficulties in communication, and poor interaction with others, which prevent them from developing skills and socially competent mechanisms (Alarcón Parco, Jó, & Patricia, 2015). Internalizing problems and lack of social skills can have long-term adverse consequences, such as school failure, difficulties in solving problems, or problems in relationships with other people (Lozano & Lozano, 2017).

## Internalizing problems and emotional intelligence

The EI concept refers to the interaction between emotion and cognition that allows individuals a functioning that adapts to their surroundings (Humphrey, Curran, Morris, Farrell, & Woods, 2007; Salovey & Grewal, 2005). For Goleman (1995), EI consists in the following: (1) knowing one’s own emotions, (2) handling emotions, (3) motivating oneself, (4) recognizing others’ emotions, and (5) establishing relationships. The idea that emotional competences are a crucial factor to explain a person’s functioning in all areas of life lies in the EI concept (Mikolajczak, Luminet, & Menil, 2006).

Nowadays, there is debate between two large conceptual perspectives: on the one hand, one that centers on basic emotional skills, based on the adaptive use of emotions by someone to solve problems and to efficiently adapt to an environment (Mayer & Salovey, 1997); on the other hand, one based on stable conduct traits and personality variables (Bar-On, 1997; Fernández-Berrocal & Extremera, 2005; Petrides, 2016).

In both approaches, the presence of internalizing problems can affect the adolescent’s emotional performance capacity, which can give rise to problems such as depression, anxiety, social anxiety, somatic complaints, and post-traumatic symptoms (Schneider, Arch, Landy, & Hankin, 2016; Shapero, Abramson, & Alloy, 2016).

## Study aims

Cross-sectional associations among internalizing problems, social skills, and EI have been reported. However, no joint prospective examinations have been made of the relationships of internalizing problems with social skills and EI. Nor are there studies that include a young adolescent sample. Based on the idea of the role that social skills and EI can play in the emergence, maintenance, and development of internalizing problems in adolescents, the objective of this research was to examine the relationship linking these three constructs (internalizing problems, EI, and social skills) in secondary education students and the role of gender in that relationship.

This study puts forward two initial hypotheses: (1) internalizing problems are related with adolescents’ social skills and EI; (2) the relationship linking these three constructs differs according to gender.

## Method

### Participants

The sample was formed by 1358 students studying compulsory Spanish secondary education (ESO), 691males (50.88%) and 667 females (49.12%), from years 1 to 4 of ESO at the public secondary education institute of Zaragoza. The participants’ ages went from 12 to 17 years, and their mean age was 14.89 years (s.d. = 1.586). This study observed the ethical considerations of the Declaration of Helsinki and met the ethical research criteria applied to human beings (voluntary participation, informed consent from mothers/fathers and students prior to the research, information rights, personal data protection and guarantees of confidentiality, no discrimination, cause-free status, and being able to drop out of the study in any of its phases). After calculating sample representativeness with a 95% confidence level and a 5% sampling error, the final surveyed sample proved representative of the province of Zaragoza. This study was designed as prospective ex post facto study (Montero & León, 2007).

### Instruments

#### SENA (System to Evaluate Children and Adolescents) (Fernández-Pinto, Santamaría, Sánchez-Sánchez, Carrasco, & del Barrio, 2015)

It comprises nine questionnaires for three age groups: infants (3–6 years), primary (6–12 years), and secondary (12–18 years). It has specific questionnaires to collect information from different informants by addressing the various settings in which children develop (self-report, family, and school). The questionnaire allows a wide series of contents to be evaluated using three problem scales (internalizing, externalizing, and contextual), a vulnerability scale, and a personal resources scale. The secondary education self-report was used in the present research, which consists of 188 items with a 5-point Likert scale. In each one, the informant evaluated the frequency with which each described conduct appeared. In the present study, work was done using the scales of internalizing problems (depression, anxiety, social anxiety, somatic complaints, post-traumatic symptoms, and obsession-compulsion), with reliability values (*α* = .90 depression, *α* = .85 anxiety, *α* = .85 social anxiety, *α* = .81 somatic complaints, *α* = .82 post-traumatic symptoms, and *α* = .68 obsession-compulsion).

#### The ICQ-15 social skills questionnaire (Buhrmester, Furman, Wittenberg, & Reis, 1988)

We used the *Spanish version of the ICQ-15* (Salavera & Usan, 2018). This scale evaluates the multidimensional construct of social skills using five different, but interrelated, scales and measures personal competence in the capacity for the following: (1) initiating relations, (2) negative assertion, (3) disclosing personal information, (4) offering emotional support, and (5) interpersonal conflict management. It comprises 15 items and takes a 5-point Likert format. The Spanish version offers high reliability (alpha = .85). Reliability in the present research was 0.84.

#### The TEIQue-SF emotional intelligence questionnaire

We used the Spanish versions of the TEIQue-SF (Petrides, 2012). This questionnaire is based on both the EI trait theory and model, within whose frame EI is deemed a personality trait, and is located at levels below the hierarchies of personality. It consists of 30 items and takes a 7-point Likert format. It has four factors: (1) well-being, (2) self-control skills, (3) emotional skills, and (4) social skills. The Spanish version offers high reliability (alpha = .83). Reliability in the present research was alpha = .81.

### Procedure

To select the sample, collaboration was requested from education centers by telephoning them. Once they confirmed their participation, a list of the participating centers was drawn up. When each scale was handed out, the participants were explained the research objectives, and the importance of answering all the items was stressed.

The participants had 30 min to complete the abovementioned scales and informed consent. Participants were reminded that any collected information would remain anonymous and confidential in all cases. The data collected in the present study were obtained in April and May 2016.

The SPSS 22.0 statistical software package was employed for the statistical analysis. Having proven the normality of the sample and the equality of variances, parametric techniques were chosen. A descriptive analysis was done for each variable. Work was conducted in all cases with the lowest possible level of significance, and any differences with a *p* < 0.05 value were taken to be significant. Contrasts were considered bilaterally. A conglomerate of means was established to obtain a cluster to classify the participants into different groups according to their internalizing problems, social skills, and EI. Finally, the AMOS v.24 statistical software was used to consider a structural equations model to validate and quantify the relationships linking internalizing problems, social skills, and EI of secondary education students.

## Results

The relationship linking internalizing problems, social skills, and EI was analyzed, as was the influence of gender (Table 1). The results showed how gender was significant in internalizing problems (depression, anxiety, social anxiety, somatic complaints, and post-traumatic symptoms), except in obsession-compulsion. Significance was also found for the well-being and self-control factors of the emotional intelligence construct and for the emotional support in social skills. This indicates differences in internalizing problems between males and females. The present research results revealed how gender mainly influenced internalizing problems, but only in some EI and social skill factors.

**Table 1:** Scores obtained in internalizing problems, social skills, and EI. Influence of gender

	Males		Females		Total		*F*	Sig.
	Mean	s.d.	Mean	s.d.	Mean	s.d.		
Depression	50.92	10.05	54.65	12.18	53.04	11.14	7.683	.006
Anxiety	46.82	9.24	53.93	10.57	50.25	10.40	34.427	.000
Social anxiety	49.65	9.12	54.04	12.12	51.64	10.83	11.684	.001
Somatic complaints	51.26	9.28	54.45	10.96	53.11	10.26	6.747	.010
Post-traumatic symptoms	49.84	10.10	53.60	12.05	51.89	11.13	7.830	.006
Obsession-compulsion	53.96	10.79	46.53	9.63	51.99	58.94	.889	.347
Well-being	30.58	6.95	29.33	7.91	30.01	7.40	5.178	.024
Self-control	26.12	5.16	23.59	6.17	25.02	5.76	25.860	.000
Emotionality	37.35	6.52	38.46	7.30	37.78	6.91	1.036	.309
Sociability	27.31	5.31	27.29	5.56	27.25	5.40	.006	.940
Initiation	10.66	2.61	10.30	2.83	10.48	2.71	.945	.332
Negative assertion	10.52	2.56	10.47	2.76	10.49	2.66	.008	.929
Emotional support	12.08	2.47	13.21	2.11	12.60	2.38	16.338	.000
Self-disclosure	10.85	2.57	11.29	3.02	11.04	2.80	.841	.360
Conflict management	10.61	2.17	10.54	2.23	10.60	2.21	.445	.505

In order to evaluate the relationship linking the three variables and in accordance with the first hypothesis, the existing correlations among the scales that measured internalizing problems, social skills, and EI were studied (Table 2). The results indicated how the internalizing problems showed an inverse relationship with EI (*r* = − .59) and social skills (*r* = − .34), which would indicate how high scores for internalizing problems would be associated with low scores for social skills and EI scales. The results highlighted how obsession-compulsion did not correlate with the other two constructs.

**Table 2:** Correlations among internalizing problems, emotional intelligence, and social skills

	Depression	Anxiety	Social anxiety	Somatic complaints	Post-traumatic symptoms	Obsession-compulsion
Well-being	− .533**	− .281**	− .373**	− .331**	− .438**	− .160*
Self-control	− .225**	− .181**	− .144*	− .203**	− .233**	.021
Emotionality	− .226**	− .059	− .249**	− .143*	− .260**	− .017
Sociability	− .198**	− .113	− .339**	− .136*	− .179**	.077
Initiation	− .181**	− .136*	− .331**	− .101	− .126	.073
Negative assertion	− .250**	− .147*	− .200**	− .095	− .140*	.073
Emotional support	− .093	.148*	.050	− .035	− .047	.050
Self-disclosure	− .299**	− .160*	− .285**	− .220**	− .245**	− .032
Conflict management	− .144*	− .039	− .074	− .110	− .138*	.013

*p < 0,05 **p < 0,01

To continue and investigate these three constructs according to gender, a cluster analysis was run (Table 3) as this is a set of the multivariate techniques used to classify a group of individuals into homogeneous groups with a marked exploratory character. We intended to find a set of groups to be assigned by different individuals by some homogeneity criterion that would allow us to define a measure of similarity or divergence to classify the research participants according to these variables: internalizing problems, social skills, and EI by gender. In turn, the gender variable was addressed to corroborate the second research hypothesis. This allowed three groups of males and three other groups of females to be formed. Males obtained higher mean scores in internalizing problems (except obsession-compulsion) and self-control. The emotional support of females scored higher than males. The mean scores obtained by males and females for the other variables were similar. The scores of both study groups were compared with the mean scores obtained in this research according to gender. With males, groups were distributed as follows: (1) formed by 291 subjects (42.11%), with lower than mean scores in internalizing problems, and higher than mean scores in social skills and EI; (2) with 64 participants (9.26%), with higher than mean levels in the three internalizing problem factors (problems with the family, school, and classmates), somewhat lower than the mean in EI and similar scores to the mean for social skills; and (3) with 336 subjects (48.63%) with higher than mean scores in internalizing problems, lower than mean scores in EI and social skills (except self-control).

**Table 3:** Cluster among internalizing problems, social skills, and emotional intelligence

	Males					Females				
	1	2	3	*x*	ds	1	2	3	*x*	ds
Depression	43.83	67.90	53.26	50.92	10.05	46.61	76.15	54.75	54.65	12.18
Anxiety	39.87	61.30	50.28	46.82	9.24	45.67	66.62	60.09	53.93	10.57
Social anxiety	43.54	61.10	52.91	49.65	9.12	45.17	66.31	61.03	54.04	12.12
Somatic complaints	44.35	67.60	54.23	51.26	9.28	46.78	70.15	58.31	54.45	10.96
Post-traumatic symptoms	41.98	67.90	53.77	49.84	10.10	46.33	73.38	54.31	53.60	12.05
Obsession-compulsion	46.70	68.00	58.21	53.96	10.79	50.19	67.46	59.47	46.53	9.63
Well-being	31.91	23.30	29.91	30.58	6.95	32.86	16.54	29.06	29.33	7.91
Self-control	27.07	22.90	24.40	26.12	5.16	24.97	21.46	24.81	23.59	6.17
Emotionality	38.85	31.10	37.02	37.35	6.52	41.22	32.69	37.53	38.46	7.30
Sociability	28.70	23.40	26.77	27.31	5.31	29.14	25.00	25.47	27.29	5.56
Initiation	11.00	9.90	10.87	10.66	2.61	11.08	9.00	9.53	10.30	2.83
Negative assertion	11.39	9.90	10.32	10.52	2.56	11.00	9.77	9.88	10.47	2.76
Emotional support	12.54	11.60	12.25	12.08	2.47	13.53	12.54	13.53	13.21	2.11
Self-disclosure	11.52	9.30	11.08	10.85	2.57	12.53	7.92	10.41	11.29	3.02
Conflict management	11.33	9.80	10.49	10.61	2.17	11.39	9.85	10.53	10.54	2.23
*N* (%)	291 (42.11%)	64 (9.26%)	336 (48.63%)			294 (44.08%)	110 (16.49%)	263 (39.43%)		

For the females, groups were made up as follows: (1) formed by 294 participants (44.08%), with lower than mean scores in internalizing problems and higher scores to the mean in EI and social skills; (2) with 110 subjects (16.49%) with higher than mean levels in internalizing problems, lower than mean scores in EI and social skills; and (3) with 263 participants (39.43%) with higher than mean scores in internalizing problems (except depression), higher scores to the mean in two EI factors (emotionality and sociability), and in three social skill factors (initiation, negative assertion, and self-revelation).

In short, 57.89% of the males obtained higher than mean scores for internalizing problems, and 9.26% of them obtained lower than mean scores for EI and social skills. Among the females, 55.92% obtained higher than mean scores in internalizing problems and lower than mean scores for their social skills and similar scores in EI.

**Fig. 1:**
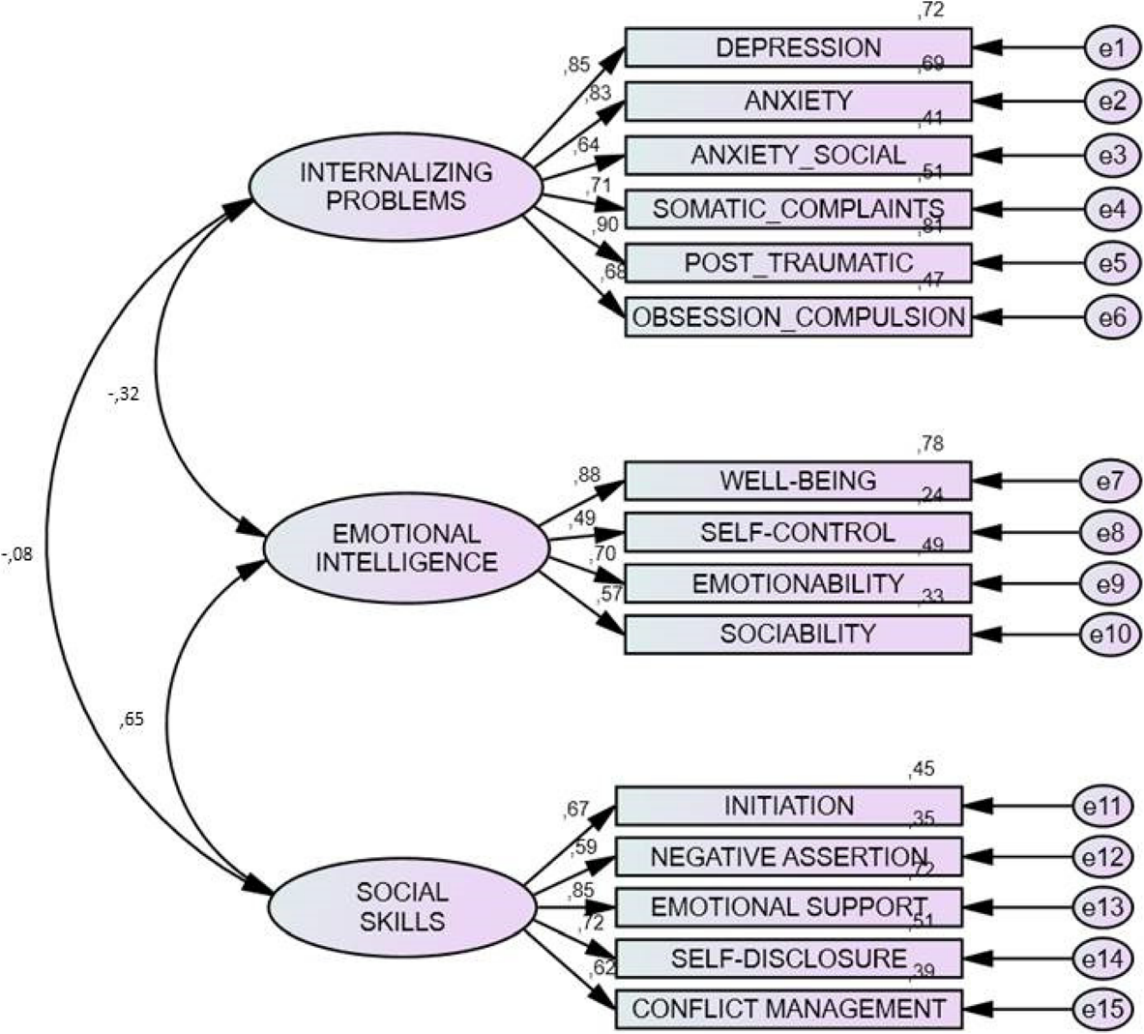
Model of the structural equations among internalized problems, social skills, and EI

Finally, an attempt was made to confirm the second hypothesis: the relationship linking internalizing problems, social skills, and EI differs according to gender. We worked with a model of structural equations. This technique combines factor analysis with linear regression to test the degree of adjustment of some observed data to a hypothesized model and expressed with a path diagram. As a result, they provide the values that belong to each relationship and, more importantly, a statistic that expresses the degree to which data conform to the proposed model to thus confirm its validity and, in this case, if the relationship linking the three research constructs differs according to gender. Figure 1 offers the analysis results with structural equations by the maximum likelihood method, which confirms the suitability of the model made up of the three constructs under study. In this case, differences were found with the analyses that considered gender: for females, the relationship between EI and social skills was stronger (*r* = .86 in females, *r* = .64 in males). Inverse relationships were found between internalizing problems and EI (*r* = −.77 in females, *r* = − .30 in males) and social skills (*r* = − .52 in females, *r* = − .07 in males). This indicates that the adolescents who obtained higher scores in internalizing problems had a lower level of EI and poorer social skills.

When we analyzed all three constructs, correlations were also observed for some gender differences. Indeed, high weight values were obtained for males for all the internalizing problem factors, which were all above .60. In females, this relationship was also strong, except for the obsession-compulsion factor (*r* = − .06). In the internalizing problems construct, differences were also found between the scores obtained by males in the anxiety and obsession-compulsion factors with higher weight values, while the depression factor obtained higher weight values in females. The relationship of the social skill factors was homogeneous with this construct, and only factors emotional support (*r* = .86 males; *r* = .61 females) and managing conflicts (*r* = .62 males; *r* = .46 females) established a stronger relationship with the social skill construct for males. In EI, males established stronger relationships in the factors well-being (*r* = .89 males; *r* = .79 females), sociability (*r* = .58 males; *r* = .42 females), and above all, self-control (*r* = .48 males; *r* = .18 females). As the various indices reflected a suitable model fit, we can state that the proposed model about the factorial structure among the three factors is sustainable: *χ*^2^(85) = 201.161 *p* < 0.001; *χ*^2^/*gl* = 2.367; CFI = 0.919; NFI = 0.869; TLI = 0.900; RMSEA = 0.075, *IC* 95% (0.062–0.089).

When nested model comparisons were made (Table 4) and when assuming the unconstrained model to be correct, we obtained a comparison made with the measurement weights, which indicated that the equal measurement weights model displayed the same fit as the model with no restrictions. Thus, the regression weights in the indicated model were the same. Likewise, the models of the measurement intercepts, structural covariances, and measurement residuals did not fit the data, and the comparison indicated that the model statistically differed and gave a worse fit. These results indicated both the gender differences and the adequacy of the model established among the three variables (internalizing problems, social skills, and EI).

**Table 4:** Multiple-group analysis of internalizing problems, emotional intelligence, and social skills by gender

Model	DF	CMIN	*P*	NFI Delta-1	IFI Delta-2	RFI rho-1	TLI rho2
Measurement weights	12	28,834	.004	.016	.018	.006	.007
Measurement intercepts	27	107,295	.000	.061	.066	.042	.047
Structural covariances	33	124,581	.000	.071	.077	.045	.051
Measurement residuals	48	729,259	.000	.413	.452	.386	.438

## Discussion and conclusions

The present work had two objectives: (1) to analyze whether internalizing problems were related with adolescents’ social skills and EI and (2) to evaluate if the relationship linking these three constructs differed according to gender.

The first hypothesis refers to a possible relationship linking internalizing problems and social skills and EI. The obtained research results corroborate this hypothesis by explaining how internalizing problems maintain an inverse relationship with social skills and EI, i.e., the higher the scores for internalizing problems, the lower the scores that adolescents obtain for performing social skills and EI, and vice versa. These results indicate how social skills and EI may act as protectors of these internalizing problems, which agrees with former research (Bubic & Ivanisevic, 2016; Martínez-Martí & Ruch, 2017; Trickey, Siddaway, Meider-Stedman, Serpell, & Field, 2012) that indicated the relationship between internalizing problems and the social competence variables. Internalizing problems (depression, anxiety, social anxiety, somatic complaints, post-traumatic symptoms, and obsession-compulsion) have been previously interrelated in recent works (Campos et al., 2014; Senzik, Shäfer, Samson, Naumann, & Tuschen-Caffier, 2017), but these works did not analyze the importance of their relationship with EI and social skills, which the present research indicates. As expected, and to a greater or lesser extent, the internalizing problem factors showed an inverse relationship with EI and social skills. However, this point presents two exceptions: somatic complaints did not correlate with social skills, and obsession-compulsion did not correlate with social skills and EI.

An attempt was made to evaluate the participants’ levels in the analyzed constructs. This work showed how two in every three of the males, and just over one in every two of the females, who participated in our research obtained higher than mean scores for internalizing problems. Generally speaking, males obtained higher scores than females for the two EI factors (well-being and self-control) and lower scores for the emotional support factor of social skills. These results differ from those obtained in former research (Ganguly, Kulkami, & Gupta, 2017; Stubbs & Maynard, 2016), which has indicated lower levels of well-being and self-control in males. However, these studies were about matters like aggression and perfectionism rather than internalizing problems, as in the present study, which might explain these results.

In order to verify the second hypothesis, we investigated if the relationship linking internalizing problems, social skills, and EI differed according to secondary education students’ gender. The model explains how social skills and EI are related inversely with internalizing problems in secondary education students and that when considering internalizing problems according to gender, a higher weight value is indicated for EI and social skills in internalizing problems for females. The present research results also indicated how the weight of the obsession-compulsion factor for females was barely present in internalizing problems (*r* = − .06), and its weight for males was similar to the other factors. Accordingly, obsession-compulsion did not correlate with either EI or social skills, an aspect which indicates that it would not fit well in the three constructs model used herein. Moreover in EI, the well-being, self-control, and sociability factors presented higher weight values in males, as did the emotional support and managing emotions factors in social skills. The present results not only back this matter, which agrees with some former studies (Burger & Samuel, 2017; Eceiza, Arrieta, & Goñi, 2008; Ferrándiz, Hernández, Bermejo, Ferrando, & Sáinz, 2012), but also fall in line with other works that have studied these variables separately, and have indicated gender differences in internalizing problems (Gomes & Soares, 2013), social skills (Zach, Yazdi-Ugav, & Zeev, 2016), and even better handling emotions among females (Balluerka, Gosostiaga, Alonso-Arbiol, & Aritzeta, 2017). However, it is worth highlighting that other works have indicated the difficulty of obtaining conclusive results along these lines (Gartxia, Aritzeta, Bulluerka, & Barberá, 2012). This reveals the necessity of further studies and research that relate these variables together according to gender, and which study this research line in depth. Moreover, there are those concepts indicated by Vantieghem, Vermeersch, and Van Houtte (2014) who point out how the theory of gender identity can be valuable for promoting gender educational research and how gender identity can be linked to certain behaviors in the classroom or study methods. Much remains to be discovered about the impact of identity on school achievement, and research is needed to promote this dichotomy and to work on the association between gender identity and internalizing problems and social skills and EI.

Despite its large sample size, this study is not without its limitations. Longitudinal-type studies that allow the evaluation of the appearance and development of internalizing problems and attach the importance of working with social skills and EI with adolescents as a preventive element of these problems are necessary. Besides, this study can and must be extended to other constructs, like affects and use of humor or personality, which should be related, and their relation with these variables should be studied.

The present results evidence a relationship among internalizing problems, social skills, and EI and, hence, the relevance of working on these aspects at school. Along these lines, this relationship indicates the need to prevent internalizing problems, which requires identifying them early. This matter can help others who live alongside adolescents (fathers, mothers, and teachers) to observe their social skills and emotional performance as they could act as ideal informers to detect such problems in adolescents.

As future perspectives, the need to implement specific education programs that work with these three constructs at school is worth stressing, particularly internalizing problems given their importance for and implication in adolescents’ development. Our research results encourage us to continue asking new questions to help us to define methodologies and to find some answers, which will allow us to make progress in building adolescents’ socio-affective development.

In conclusion, this study provides substantial empirical support for the postulated model through which internalizing problems, emotional intelligence, and social skills operate in accordance with adolescent development with the effects of EI and social skills on internalizing problems. The study also explains the gender differences in the relationship of internalizing problems, EI, and social skills. Its findings suggest the importance of promoting social skills and EI among adolescents and engaging in appropriate academic, social, and school activities.
